# A SARS-CoV-2 outbreak in a plastics manufacturing plant

**DOI:** 10.1186/s12889-023-16025-8

**Published:** 2023-06-05

**Authors:** Alice Graham, Amber I. Raja, Karin van Veldhoven, Gillian Nicholls, Andrew Simpson, Barry Atkinson, Ian Nicholls, Hannah Higgins, Joan Cooke, Allan Bennett, Derek Morgan, Chris Keen, Tony Fletcher, Neil Pearce, Christina Atchison, Elizabeth B. Brickley, Yiqun Chen

**Affiliations:** 1 Rapid Investigation Team, Field Services, UK Health Security Agency, Wellington House, London, UK; 2 Health Equity Action Lab, Department of Infectious Disease Epidemiology, London School of Hygiene & Tropical Medicine, London, UK; 3 Department of Non-communicable Disease Epidemiology, London School of Hygiene & Tropical Medicine, London, UK; 4 Science Division, Health and Safety Executive, Buxton, UK; 5 Chemical and Environmental Effects Department, UK Health Security Agency, Chilton, UK; 6 Department of Medical Statistics, London School of Hygiene & Tropical Medicine, London, UK

**Keywords:** SARS-CoV-2, COVID-19, Workplace, Outbreak, Manufacturing

## Abstract

**Background:**

A SARS-CoV-2 outbreak with an attack rate of 14.3% was reported at a plastics manufacturing plant in England.

**Methods:**

Between 23^rd^ March and 13^th^ May 2021, the COVID-OUT team undertook a comprehensive outbreak investigation, including environmental assessment, surface sampling, molecular and serological testing, and detailed questionnaires, to identify potential SARS-CoV-2 transmission routes, and workplace- and worker-related risk factors.

**Results:**

While ventilation, indicated using real-time CO_2_ proxy measures, was generally adequate on-site, the technical office with the highest localized attack rate (21.4%) frequently reached peaks in CO_2_ of 2100ppm. SARS-CoV-2 RNA was found in low levels (Ct ≥35) in surface samples collected across the site. High noise levels (79dB) were recorded in the main production area, and study participants reported having close work contacts (73.1%) and sharing tools (75.5%). Only 20.0% of participants reported using a surgical mask and/or FFP2/FFP3 respirator at least half the time and 71.0% expressed concerns regarding potential pay decreases and/or unemployment due to self-isolation or workplace closure.

**Conclusions:**

The findings reinforce the importance of enhanced infection control measures in manufacturing sectors, including improved ventilation with possible consideration of CO_2_ monitoring, utilising air cleaning interventions in enclosed environments, and provision of good-quality face masks (i.e., surgical masks or FFP2/FFP3 respirators) especially when social distancing cannot be maintained. Further research on the impacts of job security-related concerns is warranted.

## Introduction

In March 2021, an outbreak of alpha (lineage: B.1.1.7, variant: VOC-20DEC-01) SARS-CoV-2, occurred at a plastics manufacturing workplace in England, United Kingdom. An initial investigation was conducted between 17^th^ and 19^th^ March 2021 by Public Health England’s (PHE, now known as UK Health Security Agency) local Health Protection Team, finding an overall attack rate of 14.3% (33/231), with attack rates of 12.5% (2/16) in the warehouse, 12.7% (19/150) in the main production area, 20.0% (6/30) in the operations office, 21.4% (6/28) in the technical office and 0.0% (0/7) in the laboratory. After notification of this outbreak by the regional Health Protection Team (HPT) on 22^nd^ March 2021, the COVID-19 Outbreak investigation to Understand Transmission (COVID-OUT) team undertook a comprehensive investigation between 23^rd^ March and 13^th^ May 2021 to identify potential SARS-CoV-2 transmission routes, and workplace- and worker-related risk factors.

## Methods

**Fig. 1:**
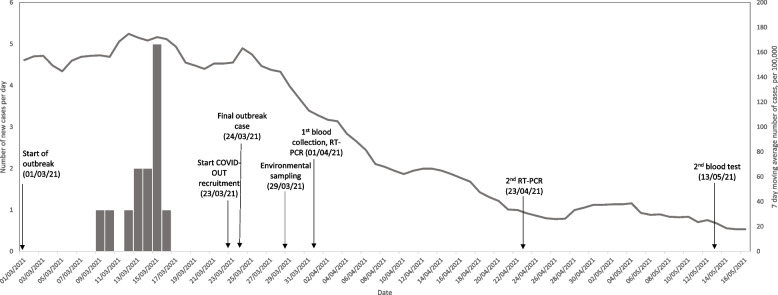
COVID-19 cases in manufacturing workplace and the surrounding region where an outbreak occurred, March 2021. Grey bars represent the number of COVID-19 cases reported by local Health Protection Teams (HPTs) associated with the plastic manufacturing company. The grey line represents the 7-day moving average number of COVID-19 cases per 100,000 in the Lower Tier Local Authority (LTLA) where the site is based. The arrows represent the timing of sampling from the COVID-OUT study

A detailed environmental assessment, following a previously described protocol for collecting site-level data on building layout, ventilation, temperature, humidity, noise levels and workforce behaviours (available at [1]), was conducted on 30^th^ March 2021. As part of the initial environmental assessment, spot measurements for carbon dioxide (CO_2_; used as a proxy for ventilation) were taken from across the site using Honeywell BW Solo monitors, and a spot measurement for noise was taken from the drinks station of the main production floor. Additionally, CO_2_ levels, temperature, and humidity were monitored between 30^th^ March 2021 to 27^th^ April 2021 in three locations (i.e., the production area, canteen and technical office) using BW Solo and Elsie logging instruments. Surface samples were collected from frequently and infrequently touched surfaces in locations across the site that were associated with confirmed cases. Samples were analysed using a two target (nucleocapsid [N] and ORF1ab) reverse transcriptase-polymerase chain reaction (qRT-PCR) (CerTest Biotec, Viasure, Zaragoza, Spain) assay to detect and quantify viral ribonucleic acid (RNA). Participant data collection included: a comprehensive baseline questionnaire and shorter follow-up questionnaires (both available at [2]), two rounds of anti-SARS-CoV-2 antibody testing (in week 1 and weeks 4-6) and three rounds of qRT-PCR testing (in weeks 1, 2, and 3) (Fig. 1). Confirmed cases were defined as participants who presented between 1^st^ March to 13^th^ May with: (i) qRT-PCR evidence of a SARS-CoV-2 infection, (ii) N-specific seroconversion, or (iii) self-reporting of a positive SARS-CoV-2 test with positive N-specific antibody results. Suspected cases were defined as participants who presented during the outbreak period with no positive qRT-PCR or N-specific antibody results in COVID-OUT testing but with: (i) self-reporting of a positive test or (ii) symptoms consistent with COVID-19 defined as: (a) acute onset of fever (>37.8C) and a new continuous cough or (b) acute onset of any three or more of fever (>37.8C), cough, shortness of breath, loss of taste or smell, runny nose, fatigue, sore throat, muscle or body aches, headache, nausea or vomiting, and/or diarrhoea [3].

## Results

**Fig. 2:**
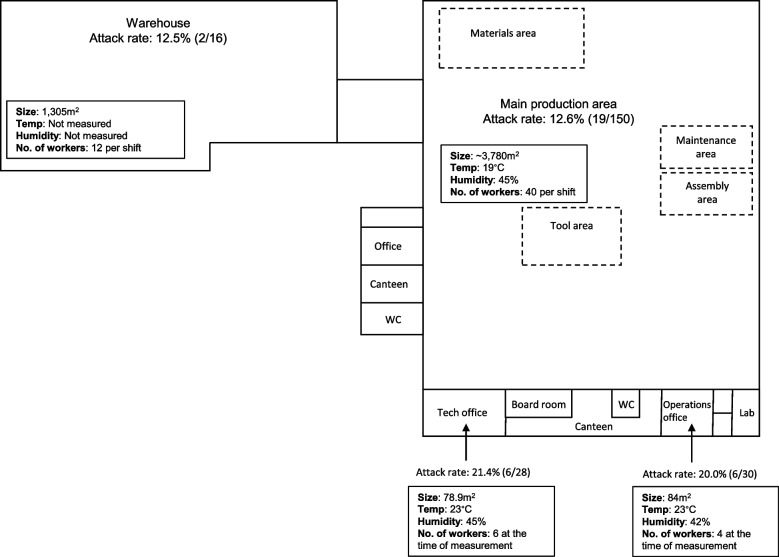
Site map and environmental assessment of a manufacturing company, where a SARS-CoV-2 outbreak occurred

The site was a one-storey building with a mezzanine area and an attached adjacent warehouse (Fig. 2). The main building comprised an open-plan production area (150 workers with approximately 40 workers per shift, three 8-hour shifts/day, 7-days of shifts, 3780 m^2^), self-contained operations office (30 workers with partial home working, 18 face-to-face desks, 84m^2^), self-contained technical office (28 workers with partial home working, 17 face-to-face desks with partial dividers, 79m^2^), two canteens, locker rooms and several meeting rooms (Fig. 2). The warehouse (12 workers per shift, 1305m^2^) was open-planned, with external doors on one side that were often open. The company reported reduced occupancy from pre-COVID-19 levels, as workers were furloughed and the number of visitors was minimized.

The manufacturing process was carried out in groups. During site visits, the COVID-OUT occupational hygienists frequently observed workers in close proximity. A spot noise reading in the manufacturing area was 79dB, suggesting workers were unlikely to be heard if a 2-meter distance was maintained, especially if face coverings were worn. Lapses in social distancing were also observed at a drink station located on the manufacturing shop floor, where face coverings were not consistently worn. Social distancing was successfully maintained in the canteen, where a maximum occupancy of five workers was implemented.

The site relied exclusively on natural ventilation, by opening windows and doors. While carbon dioxide (CO_2_) levels measured across the site, including in the main production area, were generally adequate, CO_2_ levels measured in the technical office were found to exceed the recommended 1500ppm threshold [4] 12-times over a 30 day period, with peaks up to 2100ppm that lasted several hours and corresponded to increases in daily occupancy. Out of the 66 surface samples tested, traces of SARS-CoV-2 RNA, with Ct values ranging from 36.7 to 37.9, were identified across the site, with 8 (12.1%) confirmed positives and 11 (16.7%) suspected positives (Table 1).

**Table 1:** Surface samples taken following a SARS-CoV-2 outbreak at a manufacturing workplace, March 2021, in England

**RT-PCR results** **(From a total of 66 samples)**			**Level of RNA** **(based on Ct value)**		
Confirmed Positive	Suspected Positive	Negative	Moderate-High (Ct <32.0)	Low (Ct 32.0-34.9)	Very low-None (Ct ≥35.0^a^)
8 (12.1%)	11 (16.7%)	47 (71.2%)	0 (0.0%)	0 (0.0%)	66 (%)
**Positive sample information**					
**Site area**	**Location in area**	**Mean Ct value**^b^		**Estimated copies per cm**^**2**c^	
Ops Office	Desk and Chair Arms	37.5		699	
Assembly	Desk and Under Chair	37.1		935	
Assembly	Stationery Cupboard	37.7^d^		1075	
Production	Tool Trolley	36.8		2757	
Maintenance	2x Clamps + Cupboard	37.8		329	
Tool Room	Cupboard	36.7		2641	
Tool Room	Computer	37.3		1524	
Tool Room	G Clamp Handle	37.7^d^		2206	
Production	Machinery	37.6^d^		1155	
WC	Dryer - Board Underneath	37.3^d^		922	
Canteen 2	Table and Chairs	37.3^d^		997	
Canteen 2	Leather Seat	37.4^d^		1347	
Maintenance Office	Cupboard - Handles and Inner Door	37.9^d^		882	
Warehouse	Aircon unit	36.7		918	
Warehouse	Cleaning station - tool handles	37.5^d^		103	
Locker room	Locker	37.3^d^		919	
Locker room	Front of 140, 141	37.5^d^		790	
Locker room	Bench	37.9		418	
Locker room	Bench	37.4^d^		430	

*Abbreviations*: *RT-PCR* Reverse transcription-polymerase chain reaction, *Ct* Cycle threshold, *Ops* Operations, *WC* Water Closet

^a^Includes 47 samples with no SARS-CoV-2 RNA detected

^b^Mean Ct value for the N gene

^c^Extrapolation from copies per reaction to copies per sample collected based on the dilution factor, then divided by recorded sampling area

^d^Sample identified as suspected positive, defined as a sample with a single replicate testing positive for at least one target

Among the 61 workers (male: 35/60, 58.3%; mean age, range: 38, 18-63 years) who consented to participate, 13 (21.3%) confirmed and 3 (4.9%) suspected cases were identified (Table 2). Of these, 9 (69.2%) confirmed and 2 (66.7%) suspected cases reported at least one of the following symptoms: fever, dry cough, productive cough, shortness of breath and loss of taste (Table 2). Prior to the outbreak, COVID-19 vaccination among workers was low, with only 12.7% (7/55) of participants reporting receipt of one dose.

**Table 2:** Participant data collected from a plastic manufacturing company, where a SARS-CoV-2 outbreak occurred, March 2021

**Variable**	**Categories**	**Non-cases** **n (%) (*n*=45)**	**Total cases** **n (%)** **(*n*=16)**	**Confirmed**^b^ **n (%)** **(*n*=13)**	**Suspected**^c^ **n (%)** **(*n*=3)**
RT-PCR testing^d^	Positive test^a^	0	9 (64.3)	9 (69.2)	0
	Negative test	40 (100)	5 (35.7)	4 (30.8)	1 (100)
	Missing	5	2	0	2
Anti-SARS-CoV-2 N (nucleocapsid) testing	Positive test	9 (27.3)^g^	12 (100)	12 (100)	0
	Negative test	24 (72.7)	0	0	0
	Missing	12	4	1	3
Any self-reported testing during outbreak period^e^	Positive test	0	15 (100)	12 (100)	3 (100)
	Negative test	39 (100)	0	0	0
	Missing	6	1	1	0
Sex	Male	29 (65.9)	6 (37.5)	6 (46.2)	0
	Female	15 (34.1)	10 (62.5)	7 (53.9)	3 (100)
	Missing	1	0	0	0
Age	Mean (min - max)	36 (19 - 63)	42 (18 - 60)	42 (23 - 60)	42 (18 - 57)
	Missing	1	0	0	0
Signs & Symptoms during outbreak period^e^	None reported	44 (97.8)	5 (31.3)	4 (30.8)	1 (33.3)
	Fever	0	6 (37.5)	4 (30.8)	2 (66.7)
	Productive cough	0	3 (18.8)	3 (23.1)	0
	Dry cough	0	5 (33.3)	4 (30.8)	1 (50)
	Shortness of breath	1 (2.2)	5 (33.3)	5 (38.5)	0
	Loss of taste and smell	0	8 (53.3)	7 (53.9)	1 (50)
	Missing	4	2	1	1
Pay change if participant needed to self-isolate due to COVID-19	No change	7 (17.5)	4 (26.7)	4 (33.3)	0
	Decrease / Become zero	21 (52.5)	7 (46.7)	5 (41.7)	2 (66.7)
	Don’t know	12 (30.0)	4 (26.7)	3 (25)	1 (33.3)
	Missing	5	1	1	0
Pay change if the workplace closed for two weeks due to COVID-19	No change	5 (12.5)	0	0	0
	Decrease	16 (40.0)	6 (40)	4 (33.3)	2 (66.7)
	Become zero	1 (2.5)	1 (6.7)	1 (8.3)	0
	Don’t know	18 (45.0)	8 (53.3)	7 (58.3)	1 (33.3)
	Missing	5	1	1	0
Concerns about reduced income if participant needed to self-isolate due to COVID-19	No, not at all	10 (25.0)	0	0	0
	No, not so much	8 (20.0)	3 (20)	3 (25)	0
	Yes, slightly	10 (25.0)	6 (40)	4 (33.3)	1 (33.3)
	Yes, very much	6 (15.0)	4 (26.7)	3 (25)	1 (33.3)
	Not sure	6 (15.0)	2 (13.3)	2 (16.7)	0
	Missing	5	1	1	0
Able to follow social distancing rules at the workplace and keep distance from your co-workers	Rarely	2 (5.3)	0	0	0
	Sometimes	3 (7.9)	0	0	0
	Mostly	33 (86.8)	10 (100)	9 (100)	1 (100)
	Missing	7	6	4	2
Participant needed to talk loudly or to ‘lean in’ to listen and speak to people at work	No	8 (21.1)	4 (33.3)	3 (30.0)	1 (50.0)
	Yes, sometimes	21 (55.3)	6 (50.0)	5 (50.0)	1 (50.0)
	Yes, most of the time	8 (21.1)	2 (16.7)	2 (20.0)	0
	Yes, always	1 (2.6)	0	0	0
	Missing	7	4	3	1
Facilities to wash or sanitise your hands at the workplace as often as was needed	Yes	38 (100)	12 (100)	10 (100)	2 (100)
	Missing	7	4	3	1
Close contact for social or leisure activities (such as going to pubs and restaurants)^f^	0	35 (92.1)	14 (93.3)	11 (91.7)	3 (100)
	1 to 20	3 (7.9)	1 (6.7)	1 (8.3)	
	Missing	7	1	1	0
Close contact at work^f^	0	7 (18.4)	7 (50)	4 (36.4)	3 (100)
	1 to 2	11 (28.9)	4 (28.6)	4 (36.4)	0
	3 to 20	16 (42.1)	3 (21.4)	3 (27.3)	0
	21 to 100	4 (10.5)	0	0 (0)	0
	Missing	7	2	2	2
Close contacts travelling or commuting for work^f^	0	29 (74.4)	13 (86.7)	10 (83.3)	3 (100)
	1 to 20	10 (25.6)	2 (13.3)	2 (16.7)	0
	Missing	6	1	1	0
Close contacts for essential activities (such as going food shopping or to the GP)^f^	0	17 (43.6)	10 (66.7)	7 (58.3)	3 (100)
	1 to 2	10 (25.6)	3 (20)	3 (25)	0
	3 to 100 or over	12 (30.8)	2 (13.3)	2 (16.7)	0
	Missing	6	1	1	0
Contact with a positive individual	No	17 (43.6)	2 (13.3)	2 (16.7)	0
	Yes, somebody I live with	0	5 (33.3)	4 (33.3)	1 (33.3)
	Yes, somebody I work with	8 (20.5)	4 (26.7)	3 (25)	1 (33.3)
	Unknown	14 (35.9)	4 (26.7)	3 (25)	1 (33.3)
	Missing	6	1	1	0
Other members of the household, under 20	None	9 (31.0)	3 (37.5)	3 (50)	0
	Yes, aged 0 to 11	12 (41.4)	3 (37.5)	2 (33.3)	1 (50)
	Yes, aged 12 to 19	12 (41.4)	4 (50)	2 (33.3)	2 (100)
	Missing	16	8	7	1
Shared goods or tools that had been handled by co-workers	No	9 (23.7)	3 (27.3)	2 (20)	1 (100)
	Yes	29 (76.3)	8 (72.7)	8 (80)	0
	Missing	7	5	3	2
Frequency of wearing gloves at work	Never	15 (39.5)	8 (66.7)	6 (60)	2 (100)
	Less than half the time	9 (23.7)	1 (8.3)	1 (10)	0
	More than half the time / Nearly all the time	14 (36.8)	3 (25)	3 (30)	0
	Missing	7	4	3	1
Frequency of wearing washable mask / face covering	Never	8 (21.1)	3 (27.3)	3 (33.3)	0
	Less than half the time	6 (15.8)	1 (9.1)	1 (11.1)	0
	More than half the time / Nearly all the time	24 (63.2)	7 (63.6)	5 (55.6)	2 (100)
	Missing	7	5	4	1
Frequency of wearing surgical mask / disposable mask	Never	21 (56.8)	3 (27.3)	3 (33.3)	0
	Less than half the time	4 (10.8)	4 (36.4)	3 (33.3)	1 (50)
	More than half the time / Nearly all the time	12 (32.4)	4 (36.4)	3 (33.3)	1 (50)
	Missing	8	5	4	1
Frequency of wearing mask-type FFP2 or FFP3	Never	31 (88.6)	7 (70)	7 (77.8)	0
	Less than half the time	2 (5.7)	1 (10)	1 (11.1)	0
	More than half the time / Nearly all the time	2 (5.7)	2 (20)	1 (11.1)	1 (100)
	Missing	10	6	4	2
Employment contract	Permanent	39 (92.9)	13 (86.7)	11 (91.7)	2 (66.7)
	Less than a year fixed	2 (4.8)	1 (6.7)	1 (8.3)	0
	Zero hours contract	0	1 (6.7)	0	1 (33.3)
	Other	1 (2.4)	0	0	0
Vaccination prior to the outbreak	Missing	3	1	1	0
	1^st^ dose	6 (14.3)	1 (7.1)	1 (9.1)	0
	2^nd^ dose	0	0	0	0
	Missing	3	2	2	0
Vaccine type	Pfizer / BioNTech	2 (33.3)	1 (100)	1 (100)	0
	Oxford / AstraZeneca	4 (66.7)	0	0	0
BMI category	Normal weight (18.5 to 24.9)	19 (50.0)	4 (25.0)	4 (30.8)	0
	Overweight (25.0 to 29.9)	11 (28.9)	6 (37.5)	5 (38.5)	1 (33.3)
	Obese (30+)	8 (21.1)	3 (18.8)	2 (15.4)	1 (33.3)
	Missing	7	3	2	1
Smoking status	No, never	17 (41.5)	10 (71.4)	8 (66.7)	2 (100)
	No, ex-smoker	7 (17.1)	2 (14.3)	2 (16.7)	0
	Yes, currently	17 (41.5)	2 (14.3)	2 (16.7)	0
	Missing	4	2	1	1
Shared meeting rooms	None	17 (37.8)	3 (27.3)	1 (11.1)	2 (100)
	Yes, co-workers only	21 (55.2)	8 (72.7)	8 (88.9)	0
	Missing	7	5	4	1
Shared lockers	None	19 (50.0)	8 (66.7)	6 (60)	2 (100)
	Yes, co-workers only	19 (50.0)	4 (33.3)	4 (40)	0
	Missing	7	4	3	1
Shared car	None	35 (97.2)	11 (100)	9 (100)	2 (100)
	Yes, members of the public	1 (2.8)	0	0	0
	Missing	9	5	4	1
Shared canteen	None	7 (18.4)	3 (25)	2 (20)	1 (50)
	Yes, co-workers only	29 (76.3)	9 (75)	8 (80)	1 (50)
	Missing	9	4	3	1

*Abbreviations*: *COVID-19* Coronavirus disease 2019, *Ig* Immunoglobulin, *RT-PCR* Reverse transcription-polymerase chain reaction, *BMI* Body Mass Index, *FFP* Filtering Face Piece, *GP* General Practitioner

^a^Five out of 61 non-cases did not undertake any testing as part of COVID-OUT and so are not confirmed negatives but classified as non-cases

^b^Confirmed cases were defined as participants who presented during the outbreak period with: (i) RT-PCR evidence of a SARS-CoV-2 infection, (ii) N-specific seroconversion, or (iii) self-reporting of a positive test (i.e., by RT-PCR or LFD) with positive N antibody results.

^c^Suspected cases were defined as participants who presented during the outbreak period with no positive RT-PCR or N antibody results in COVID-OUT testing but with: (i) self-reporting of a positive test (i.e., by RT-PCR or LFD) or (ii) symptoms consistent with COVID-19 defined as: (a) acute onset of fever (>37.8C) and new continuous cough or (b) acute onset of any three or more of fever (>37.8C), cough, shortness of breath, loss of taste or smell, runny nose, fatigue, sore throat, muscle or body aches, headache, nausea or vomiting, and/or diarrhoea.

^d^Individuals lost to follow up (9 individuals for 1^st^ PCR, 26 individuals for 2^nd^ PCR, 34 individuals for 3^rd^ PCR)

^e^Outbreak period: 1^st^ March to 17^th^ March

^f^‘Close Contact’ defined as typically spending more than 15 minutes within 2 metres of someone

^f^Categories not mutually exclusive

^g^According to the pre-defined case definition individuals with a positive anti-SARS-CoV-2 N (nucleocapsid) tests had to also self-report a positive SARS-CoV-2 test to be classified as a case

Most participants reported having received on-site COVID-19 training (50/57, 87.7%), and cases and non-cases reported generally similar contact patterns and COVID-19 protective measures (Table 2). While 98.1% (51/52) of workers reported wearing some type of face covering (i.e., including face shields made in-house and reusable masks) at least half the time, only 20.0% reported using either FFP2/FFP3 (4/45, 8.9%) or surgical/disposable (16/48, 33.3%) masks more than half the time (Table 2). While 89.6% (43/48) of participants reported social distancing from colleagues most the time, a majority reported having to lean-in when talking to colleagues (38/50, 76%) and having close contacts (i.e., spending ≥15 minutes within 2 meters) at work (38/52, 73.1%), although most (22/37, 59.5%) close work contacts were for <1-hour. Participants also reported sharing tools (37/49, 75.5%), lockers (23/50, 46.0%), and the canteen (38/48, 79.2%) with other workers, but all reported access to hand sanitising facilities (50/50, 100%) (Table 2). While most participants reported being on a permanent contract (52/57, 91.2%), 71.0% (39/55) expressed having concerns regarding losses of income and/or employment due to workplace closure or self-isolation (Table 2), and 22.2% (12/54) reported knowing of at least one positive work contact.

Few participants reported close contacts during social events (4/53, 7.5%) or while commuting (12/54, 22.2%). While no participants reported car sharing with colleagues (0/46, 0%), one case and three non-cases (4/50, 8.0%) reported living with someone they worked with. Both confirmed (4/12, 33.3%) and suspected (1/3, 33.3%) cases reported living with someone who tested positive for COVID-19, although the timing of intra-household cases is uncertain (Table 2).

Prior to the outbreak, the company’s infection control measures included rapid SARS-CoV-2 antigen testing by lateral flow device of all workers every 7 to 10 days and provision of sanitising facilities, social distancing guidance posters on meeting rooms, and once-per-shift cleaning. Following the outbreak, the company increased SARS-CoV-2 lateral flow testing to twice per week, provided surgical face masks, face shields, and portable hand sanitisers, improved ventilation through more frequent opening of doors and windows (although, all trickle vents in the windows in the offices and canteen were closed at the time of the COVID-OUT environmental assessment), introduced a weekly electrostatic surface sanitising service, and encouraged office workers to work from home. Additionally, the Local Authority introduced a temporary on-site mobile RT-PCR testing facility. Vaccination rates continued to improve after the outbreak, with a further 23.2% (13/56) workers reporting having received a COVID-19 vaccine from 17^th^ March 2021 to 13^th^ May 2021.

## Discussion

In March 2021, a plastics manufacturing workplace in England experienced a cluster of SARS-CoV-2 cases, initially affecting 14.3% of the workforce, which was significantly higher than the cumulative incidence in the local area during the same period (p<0.0001, Chi-squared, Fig. 1). The high attack rates across different work areas as well as the broad distribution of SARS-CoV-2-positive surface samples, provide evidence of widespread viral shedding throughout the workplace. While the UK was under its third national lockdown during this outbreak, essential work sectors, which employ an estimated 53.4% of UK workers [5], continued to operate with varying degrees of on-site attendance. Whereas only 7.5% of participants reported social contacts outside of work during the study period, 71.3% of participants reported close contacts while at work. Manufacturing workplaces have been particularly susceptible to SARS-CoV-2 outbreaks, as opportunities for homeworking are limited and social distancing, especially along production lines, can present challenges. An analysis of 199 workplace outbreaks from the first wave of the pandemic in Ontario, Canada, found the manufacturing sector experienced both the largest proportion (44.7%) of workplace outbreaks as well as the largest scale outbreaks [6].

As COVID-19 vaccines were not widely available to younger ages in England at this time, the workplace primarily relied on non-pharmaceutical control measures to prevent COVID-19 cases among its staff. Of note, on-site ventilation was provided naturally (i.e., rather than mechanically), where its effectiveness is dependent on external environmental factors like humidity, wind speed, and temperature and internal factors like occupancy and window opening behaviours [7]. Although CO_2_ proxy measures generally did not exceed the recommended threshold of 1500ppm, we note that the highest attack rate of 21.4% was found in the technical office, which had both peaks in CO_2_ levels up to 2100ppm and the highest relative worker occupancy levels of 2.2 workers per 10m^2^. This finding aligns with recent models that suggest inadequate ventilation may contribute to both far-field and within-room inhalation transmission of SARS-CoV-2 [8]. Although it is well-demonstrated that effective ventilation can reduce risk of far-field inhalation transmission, settings where there is no mechanical ventilation, as is the case for this workplace, and natural ventilation cannot reliably supply sufficient fresh air, suitable portable air cleaning interventions such as HEPA (high-efficiency particular air) filters and/or UV (ultraviolet) radiation air disinfection could be used to remove or deactivate potential viruses from the air [9, 10]. Evidence suggests a reduction in the detection of SARS-CoV-2 in air samples following the use of HEPA air filtration [11], as well as the augmentation of airborne viral elimination when combined with primary mechanical ventilation methods [12]. However, further studies are required to assess the effects of portable HEPA air filters on the incidence of infections [13]. In addition, only 20.0% of participants reported surgical mask and/or FFP2/FFP3 respirator use on-site. In the time since the outbreak, mounting evidence (e.g., [14, 15]) has been published that suggests that face coverings provide a gradient of protection against SARS-CoV-2 infection risks in indoor settings, with the lowest protection provided by face shields and reusable face coverings.

A key finding of the investigation is the high frequency of workers with concerns regarding risks of pay decreases/or unemployment (71.0%) as a result of self-isolation or workplace closure. Preliminary evidence demonstrates that job insecurity due to COVID-19 and financial concerns are associated with greater depressive and anxiety symptoms [16]. Further research is warranted to discern the extent to which these concerns may have driven ‘presenteeism’ and work attendance despite having symptoms consistent with SARS-CoV-2.

Fomite transmission of SARS-CoV-2, such as through the reported frequent sharing of tools (75.5%), cannot be fully excluded, although we note that all SARS-CoV-2 RNA levels recovered from surfaces were found to be at very low quantities (Ct ≥35). This may reflect low amounts of virus shed, viral degradation over time or due to cleaning, as regular hygiene protocols were implemented prior to the outbreak. Evidence from a 2021 systematic review of SARS-CoV-2 surface contamination found the presence of SARS-CoV-2 RNA, which was extremely sensitive to detergents and disinfectants, on a wide range of surfaces; however, to date, there is no evidence of viable virus on these surfaces [17].

Overall, this study adds to the limited body of published evidence describing SARS-CoV-2 outbreaks in the non-food manufacturing sector and highlights the particular vulnerability of high-occupancy and/or enclosed offices within these workplaces. The main limitation of this investigation was the 26.4% participation rate, which included an overrepresentation of female workers compared to the whole workforce (41.7% vs. 29.2%). Along with social desirability biases, gender-related differences in COVID-19 preventive health behaviours, as previously reported [18] may have also resulted in overestimates in the reported uptake of infection control measures in the current study. The small sample size also precluded meaningful investigation of risk factors between cases and non-cases, highlighting the importance of conducting pooled analyses across workplaces in the future. Another limitation of the investigation is that the environmental assessment started five days after the last case from this site was confirmed by the local health protection teams. If surface sampling was performed closer to the peak of the outbreak, this would have enabled more meaningful interpretation of SARS-CoV-2 RNA levels.

Given the nature of work in the manufacturing sector (e.g., with unavoidable close contact for extended periods of time on production lines, particularly when sound levels are high), respiratory infectious diseases can spread rapidly even when community transmission is relatively low. As previously noted [3], the manufacturing sector may also benefit from tailored infection prevention and control guidance including, for example, specific recommendations regarding layered preventive measures and active monitoring approaches. Overall, the findings of the current study reinforce the importance of enhanced infection control measures, including improved ventilation with potential consideration of CO_2_ monitoring, utilising air cleaning interventions (e.g., HEPA filters) in enclosed environments and provision of good-quality face masks (i.e., surgical masks or FFP2/FFP3 respirators), especially when social distancing cannot be maintained. These control measures should be considered in the prevention of future outbreaks in the manufacturing sector. Further research on the impacts of job security-related concerns as well as sick leave policies and their communication is needed.

## Data Availability

The data used in this study was collected by the COVID-OUT study team, and therefore, is available from the corresponding author on reasonable request.
